# Probing of New Polymer-Based Microcapsules for Islet Cell Immunoisolation

**DOI:** 10.3390/polym16172479

**Published:** 2024-08-30

**Authors:** Polina Ermakova, Ekaterina Vasilchikova, Maxim Baten’kin, Alexandra Bogomolova, Alexey Konev, Natalia Anisimova, Alena Egoshina, Mariya Zakharina, Julia Tselousova, Nasipbek Naraliev, Denis Kuchin, Liya Lugovaya, Vladimir Zagainov, Sergey Chesnokov, Aleksandra Kashina, Elena Zagaynova

**Affiliations:** 1 Federal State Budgetary Institution of Higher Education, Privolzhsky Research Medical University, Ministry of Health of Russia, 603082 Nizhny Novgorod, Russia; vasilchikova_ea@pimunn.net (E.V.); bogomolova_a@pimunn.net (A.B.); tselousova.julia@yandex.ru (J.T.); nasip_95_kg@mail.ru (N.N.); pomc.kuchin@gmail.com (D.K.); liya.lugovaya@inbox.ru (L.L.); zagainov@gmail.com (V.Z.); meleshina_a@pimunn.net (A.K.); ezagaynova@gmail.com (E.Z.); 2 Federal State Educational Institution of Higher Educational Institution “National Research Nizhny, Novgorod State University Named after N.I. Lobachevsky”, 603105 Nizhny Novgorod, Russia; 3 Federal State Budgetary Institution of Science Institute of Organometallic Chemistry, G.A. Razuvaev Russian Academy of Sciences, 603950 Nizhny Novgorod, Russia; batenkinmax@iomc.ras.ru (M.B.); alex-kon@mail.ru (A.K.); nata.d.anisimova@yandex.ru (N.A.); lokteva@iomc.ras.ru (A.E.); m.zakharina@mail.ru (M.Z.); sch@iomc.ras.ru (S.C.); 4 Nizhny Novgorod Regional Clinical Hospital Named after N.A. Semashko, 603005 Nizhny Novgorod, Russia; 5 State Budgetary Healthcare Institution “Nizhny Novgorod Regional Clinical Oncology Dispensary”, 603163 Nizhny Novgorod, Russia; 6 Federal Scientific and Clinical Center for Physico-Chemical Medicine Named after Academician Yu. M. Lopukhin, 119334 Moscow, Russia

**Keywords:** microcapsules, microencapsulation, alginate, islet of Langerhans

## Abstract

Islet allotransplantation offers a promising cell therapy for type 1 diabetes, but challenges such as limited donor availability and immunosuppression persist. Microencapsulation of islets in polymer-coated alginate microcapsules is a favored strategy for immune protection and maintaining islet viability. This study introduces Poly [2-(methacryloyloxy)ethyl]trimethylammonium chloride (PMETAC) as an innovative coating material for microcapsules. PMETAC enhances biocompatibility and durability, marking a significant advancement in islet encapsulation. Our approach combines alginate with PMETAC to create Langerhans islet microcapsules, simplifying material composition and preparation and ultimately lowering costs and increasing clinical applicability. Our comprehensive evaluation of the stability (including osmotic stability, thermal stability, and culture condition stability) and cytotoxicity of a novel microencapsulation system based on alginate-PMETAC-alginate offers insights into its potential application in islet immunoisolation strategies. Microcapsules with PMETAC content ranging from 0.01 to 1% are explored in the current work. The results indicate that the coatings made with 0.4% PMETAC show the most promising outcomes, remaining stable in the mentioned tests and exhibiting the required permeability. It was shown that the islets encapsulated in this manner retain viability and functional activity. Thus, alginate microcapsules coated with 0.4% PMETAC are suitable for further animal trials. While our findings are promising, further studies, including animal testing, will be necessary to evaluate the clinical applicability of our encapsulation method.

## 1. Introduction

Islet allotransplantation is a promising cell therapy for the treatment of type 1 diabetes. To date, islet cells have been successfully transplanted into patients in clinical trials around the world. However, scaling up the islet transplantation technology to the general population of patients with type 1 diabetes remains a major challenge [1]. The issue is that islet transplantation is limited by the shortage of organ donors, islet death after transplantation, and the need for lifelong immunosuppression to prevent immune rejection [2]. The need for immunosuppressant use is a major concern as they can cause side effects such as kidney dysfunction, increased susceptibility to infections, and an increased risk of cancer. Additionally, immunosuppressive drugs may also have deleterious effects on the transplanted islets themselves, which in turn may lead to transplant rejection [3]. Successful islet encapsulation can eliminate immunosuppressant use. The biocompatible, semi-permeable microcapsules allow the diffusion of oxygen, insulin, and nutrients while blocking immune cells. They must selectively permit low-molecular weight compounds and address challenges in engraftment, survival, rejection, and islet homeostasis [2,4]. 

There are three approaches to encapsulating pancreatic islets: macro-, micro-, and nano-encapsulation. Microencapsulation is the preferred approach for pancreatic islet transplantation as such microcapsules provide an optimal surface-to-volume ratio that aids the fast exchange of nutrients and hormones [5]. The most common approach to the microencapsulation of pancreatic islets is the use of microcapsules based on polymer-coated alginate [6]. The most frequently used polymers are poly-L-lysine (PLL) and poly-L-ornithine (PLO) [7]. Although these capsules have been extensively studied, they do not meet the parameters for biocompatibility. The use of such capsules causes an inflammatory reaction to the transplant [8,9]. A range of other alginate-based systems have been explored, including ultra-high-viscosity alginate, Alg-cellulose sulfate)-poly(methylene-coguanidine), Alg-chitosan, Alg-PLL-poly(acrylic acid), Algpoly(L-ornithine)-Alg, Alg-PLL-poly(ethylene glycol), Algchitosan-poly(ethyleneglycol), and Alg-PLL-poly(ethyleneglycol)-Alg. Problems still facing the survival of encapsulated islets include immune rejection, death due to lack of nutrients, post-transplantation hypoxia, and loss of microcapsule stability [10]. Survival after transplantation can be increased by improving the encapsulation technologies and through searches for new biocompatible materials and their combinations [11]. Such microcapsules should have good biocompatibility, stable structural and mechanical properties, and be selectively permeable [12].

**Figure 1:**
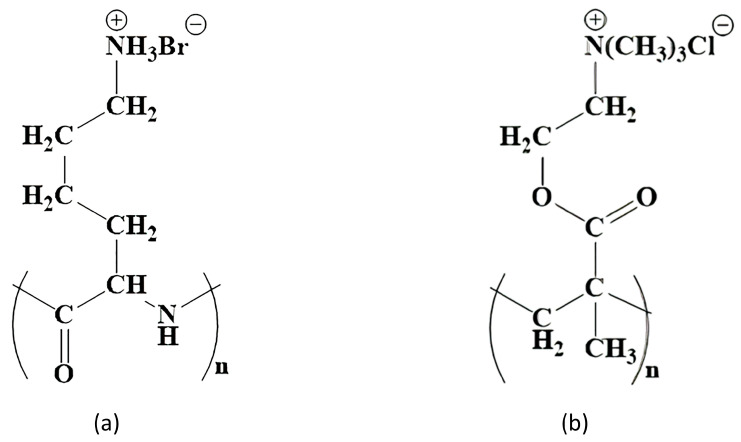
Similarities in the structure of polymer molecules (**a**) PLL and (**b**) PMETAC.

Potential polymer candidates for this include poly [2-(methacryloyloxy)ethyl]trimethylammonium chloride (PMETAC). PMETAC has a similar structure to PLL and is cationic, which is a necessary condition for the formation of the layer on the surface of the alginate microcapsule. Both polymers have fragments of the same length between the polymer chain and the nitrogen atom (Figure 1).

In addition to there being an effective synthetic route and its sound physico-chemical characterization, the safety of PMETAC should be considered. The absence of a cytotoxic effect of PMETAC has already been evaluated using three different cell-based assays (MTT, Neutral Red, and LIVE/DEAD^®^) and relevant immune cells, including two mouse cell lines (J774A.1 and BV2), as well as human peripheral blood mononuclear cells (PBMCs) [13]. The use of PMETAC as a biocompatible material has also been proposed previously. It is known that modification of the surface of poly(etheretherketone) with PMETAC leads to a significant improvement in blood compatibility and reduces pericapsular fouling around the polymer implant [14]. Furthermore, the inclusion of this polymer in a complex with poly(2-hydroxyethyl methacrylate) (HEMA) and fibroblast growth factor (bFGF) has been found to promote the regeneration of nervous tissue and functional recovery after spinal cord injury in rats [15]. In another study, recipient rat axons, astrocytes, and blood vessels grew into an implant based on the NEMA–PMETAC complex, together with neural progenitor cells (iPSC-NPs), when transplanted into an area of spinal cord injury. This polymeric complex integrated into the injured spinal cord, reduced cavity formation, and maintained iPSC-NP cell survival [16]. PMETAC has also been found to have improved biocompatibility and is a promising coating for medical devices [17]. Previously, when PMETAC was used as a copolymer in the creation of alginate microcapsules for the encapsulation of C2C12 mouse cells, it was shown that the permeability of the obtained microcapsules had a cutoff at 70 kDa, potentially making these suitable for the encapsulation of islets. Shen et al., 2009 have described the possible formation of a polyelectrolyte complex between PMETAC and negatively charged proteins (e.g., albumin). Fortunately, the relatively simple measure of replacing the regular medium with a serum-free medium for incubation after fabrication eliminated any host reactions [18]. We took this experience into account and synthesized a new type of capsule that has not been used before. Moreover, the PMETAC polymer has not previously been used to encapsulate the islets of Langerhans to compensate for insulin deficiency.

Thus, we consider PMETAC to represent a simple and stable alternative to poly-L-lysine and propose it as a potential polymer coating for microcapsules for pancreatic islet encapsulation. Such microcapsules could provide higher biocompatibility and graft stability. Moreover, we believe that the new capsule based on alginate and PMETAC has the potential for widespread use in clinical practice to compensate for insulin deficiency conditions. Our work is the first time that a comprehensive analysis has been conducted into the stability (osmotic stability, thermal stability, and stability under culture conditions) and cytotoxicity of a new microencapsulating system based on alginate-PMETAC-alginate for potential future use in islet immunoisolation.

## 2. Materials and Methods

### 2.1. Animals

The pancreatic source material was obtained from 12–15-week-old Wistar rats. All our studies with experimental animals were approved by the local ethics committee of the Privolzhsky Research Medical University (protocol No. 10; date: 26 June 2020).

### 2.2. Islet Isolation

Perfusion of the pancreas was performed using a solution of collagenase V (Collagenase from Clostridium histolyticum, Sigma, Saint Louis, MI, USA) in a modified Hanks solution enriched with CaCl_2_. Then, the perfused pancreas was shaken on a shaker preheated to 37 °C for 11–15 min. During digestion, the pancreatic tissue gradually softened until the whole organ dispersed into small granules. The digestion was stopped by adding HBSS supplemented with 5% BSA. Purification of the OLs from the exocrine tissue was carried out by triple filtration through a metal sieve with a mesh diameter of 0.5 mm. Any remaining undigested tissue was then discarded. The suspension was centrifuged at 200× *g* for 3 min and the supernatant was removed. The pellet was then centrifuged on density gradients of Ficoll DL-400 (1.095, 1.084 and 1.072, 1.048 mg/L) (Sigma, Saint Louis, MI, USA) at 800× *g* for 15 min. After density gradient centrifugation, the islets were washed with MHBS. The isolated OLs were maintained in RPMI (Gibco, London, UK) culture medium with a low glucose level supplemented with L-glutamine (0.58 mg/mL) (PanEco, Moscow, Russia), 10% FBS (Gibco, London, UK), and antibiotic-antimycotic (Antibiotic-Antimycotic 100X (ThermoScientific, Waltham, MA, USA)) at 37 °C and 5% CO_2_. During isolation, dithizone staining was performed to identify the islet cells. Staining was visualized using a Leica DM2500 microscope (Leica, Berlin, Germany).

### 2.3. Polymer Synthesis

Poly [2-(methacryloyloxy)ethyl]trimethylammonium chloride (PMETAC) was synthesized by polymerization from [2-(methacryloyloxy)ethyl]trimethylammonium chloride (Aldrich, 408107, Saint Louis, MI, USA) according to the procedure described in [19].

The IR spectrum of the polymer was recorded using an FT 801 IR Fourier spectrometer with an ATR attachment (OOO NPR Simeks, Novosibirsk, Russia). The polymer was placed on the diamond substrate of the ATR attachment and compressed with the built-in press. Determination of the molecular weight distribution of the polymer samples was carried out by gel permeation chromatography using a high-performance liquid chromatograph, the LC-20AD (Shimadzu, Kyoto, Japan). Analysis conditions: eluent 0.5 N acetic acid solution, flow rate 0.8 mL/min, Т = 30 °С, an ELSD detector (low-temperature evaporative light scattering detector). Column: TSK-GEL G3000SWXL, 7.8 mm ID × 30.0 cm L, 5 µm.

### 2.4. Microcapsule Synthesis

To conduct research on the development of the alginate microcapsules, a microfluidic installation (microfluidic device) was developed for the controlled formation of microdroplets, using an external influence on the flow of the continuous phase (Figure 1). The microfluidic setup is protected by know-how order No. 249/Akhd (dated 09/02/2022). Prior to the experiment, the microfluidic device was cleaned with alcohol and UV-sterilized for 15 min, and all solutions for use were aseptically processed. When forming microcapsules that did not contain pancreatic islets, a low-viscosity solution of sodium alginate («Aldrich», A1112, Saint Louis, MI, USA) (2 wt.%) and dextran of molecular weight 20,000 Da (АО «Vector», Novosibirsk, Russia) (15 wt.%) in phosphate-buffered saline was used as the dispersed phase (material for future microcapsules). During the formation of the microcapsules containing pancreatic islets, the concentration of sodium alginate and dextran was 2.7 and 20 wt.%, respectively. Prior to encapsulation, a suspension comprising 1 part pancreatic islets in a nutrient medium was combined with 3 parts of the alginate composition, resulting in a volumetric ratio of 1:3. The concentration of islets in the final suspension for encapsulation was 15 thousand/mL. To visualize the process of microcapsule formation, the dispersed phase was stained with methylene blue (Khimreaktiv, Russia). A solution of polyethylene glycol of molecular weight 8000 Da (PEG-8000) (Nizhnekamskneftekhim, Russia) (30 wt.%) in distilled water was used as the continuous phase.

The resulting microcapsules were placed in a chloride gelling solution of BaCl_2_·2Н_2_O (Khimreaktiv, Russia) (2.9 wt.%). Then, the gelled hydrogel microcapsules were washed with a buffer solution based on Tris (Aldrich, Saint Louis, MI, USA) (0.45 wt. %), pH = 7.2. After that, to increase the stability and to ensure the desired permeability, the microcapsules were incubated in a solution of poly [2-(methacryloyloxy)ethyl]trimethylammonium chloride in buffer solution for 10 min. Next, the microcapsules were again washed in buffer solution. Then, to increase the biocompatibility of the microcapsules, an additional alginate shell was applied to the surface of the polymer membrane by incubating them in a 0.2 wt.% solution of sodium alginate in physiological saline. Finally, the microcapsules were washed in buffer solution and placed in Hank’s solution (HBSS) (PanEko, Moscow, Russia).

### 2.5. Permeability Measurements

We evaluated the effectiveness of the сapsules at preventing penetration by macromolecules while permitting that of insulin, glucose, and other low-molecular weight molecules. Effective immunoisolation requires that the hydrogel layer excludes immunoglobulin G (IgG) and the complement molecule C3—both of which mediate the recognition of foreign cells by the immune system. These molecules are approximately 180 and 185 kDa in size, respectively.

The permeation test was carried out at 4 °C following established protocols [20,21,22]. For characterization of their permeability, different microcapsules were incubated with FITC-coupled lectins of a range of molecular weights. Lectins bind to carbohydrates found on the surfaces of islet cells. The microcapsules were incubated for 48 h at 4 °C in 0.5 mL of the following lectin solution: 5 µL (10 mg/µL) FITC-Triticum Vulgare (WGA, molecular weight 36 kDa), 14 µL (2 mg/µL) FITC-Maackia amurensis I (MAL-I, molecular weight 75 kDa), 5 µL (10 mg/µL) FITC Ricinus communis (RCA-I, molecular weight 120 kDa), and FITC-Sambuca nigra (SNA, molecular weight 150 kDa). After incubation, the microcapsules were washed 3 times with HBSS solution and examined microscopically. Image analysis was performed with ImageJ 1.43u. The number of fluorescent lectins that had penetrated the microcapsules was counted over an area of 10,000 pixels^2^ in the brightest part of the image.

### 2.6. Evaluation of Osmotic Stability

The resistance of the microcapsules to deformation and their elastic properties were determined using an osmotic pressure test. The test was performed according to the protocol described in Verheyen 2019. In this test, the microcapsules were subjected to sequential exposure to H_2_O (2 h, 0 mOsm) and HBSS++ (15 min, 24 h, and 72 h, 270–305 mOsm). Microcapsule resistance to swelling was defined by the maximum deformation attained at the end of the osmotic stress period. Microcapsule elasticity was determined by the diameter recovery achieved after the stress was removed. The tests were carried out at 2 and 7 days after manufacture.

### 2.7. Evaluation of Thermal Stability

Before testing, the diameters of each group of microcapsules in the HBSS solution were measured. Next, the microcapsules were placed under temperature conditions (37 °C and 40 °C). To study the dynamics of changes, measurements of microcapsule sizes were carried out after 3 and 7 days. The HBBS solution was replaced every day.

### 2.8. Stability of Microcapsules under Culture Conditions

To study the stability of the microcapsules under cell culture conditions, they were placed in an RPMI culture medium containing 10% fetal bovine serum (FBS) and an antimycotic antibiotic at 37 °C in a 5% CO_2_ atmosphere. Microcapsule sizes were measured before the start of the experiment and after 7 days.

### 2.9. Stability of Microcapsules under Exposure to Saline

Assessment of the stability of the alginate microcapsules was carried out by repeated washing with saline solution in a 2 mL syringe. The initial number of microcapsules used was 4500. The microcapsules were deposited, then the volume they occupied was recorded, after which excess fluid was removed. After that, physiological saline was drawn into the syringe to a volume of 2 mL. The microcapsules in the resulting solution were mixed and kept for 5 min, then the process was repeated. Saline solution was used for this test since it contains sodium ions that can replace the cross-linking ions of the alginate microcapsule.

### 2.10. Viability and Functional Activity of Encapsulated Islets

To identify the morphology and insulin synthesis of islets before and after encapsulation, we used dithizone staining (DTZ).

The survival or death of isolated islet cells was assessed using the Live/Dead Cell Double Staining Kit (Sigma-Aldrich, Saint Louis, MI, USA) at 24 h after isolation and after encapsulation, according to the standard protocol. A 500 μL sample of an islet suspension was incubated in a mixture of 2 μL calcein-AM (live cell, green) and 1 μL ethidium homodimer-1 (dead cell, red) for 15 min at room temperature. Visualization was performed on an LSM 880 confocal fluorescence microscope (Carl Zeiss, Mainz, Germany). For calcium-AM detection, fluorescence excitation at 488 nm was used, with detection in the range of 500–549 nm; propidium iodide fluorescence was excited at 543 nm, with detection in the range of 611–700 nm. The ratio of the green-colored area to the total cell area (%) was calculated from the confocal images of the aggregates in 3–5 randomly selected fields per sample using ImageJ software.

For functional activity studies, samples containing 350 islets/mL each were incubated at 37 °C and 5% CO_2_ for 24 h in cultural medium in standard conditions. The supernatant was then collected and stored at −20 °C for analysis. The concentration of the insulin released during incubation was detected using an insulin enzyme-linked immunosorbent assay (Cloud-clonecorp, Houston, TX, USA). Absorbance was measured using a Microplate reader with a 450 nm wavelength filter and expressed as (μg/mL). An assay was performed on non-encapsulated islets to serve as a control for the assay on the encapsulated islets.

### 2.11. Statistical Analysis

The permeability studies were performed in 2 independent tests, each of which analyzed at least 10 images. The stability studies involved 3 independent tests, each divided into 3 technical replicates, with 50 microcapsules from each repetition being analyzed; thus, the total number of microcapsules analyzed for each time point and each microcapsule variant was 450. The data are plotted as the medians ± the 25th and 75th quartiles. The distribution of all the data was first checked for normality using the Kolmogorov–Smirnov and Shapiro–Wilk tests. Statistical analysis of islet cell data was performed using the Mann–Whitney U test.

## 3. Results

### 3.1. Characterization of PMETAC Polymer

The synthesized PMETAC cationic polymer has an average molecular weight of Mw = 450 kDa, with a polydispersity coefficient of Mw/Mn = 1.4.

FTIR spectrometry of the PMETAC showed the following absorption bands, ν/cm^–1^: 1720 (s) (C=O); 1477 (c) (C-H); 1233 (Wed) (-COO); 1143 (c) (C-O-C); 897 (w) (C-C, C-H); 618 (w) (C-C, C-H); and 537 (w) (C-C, C-H).

### 3.2. Microcapsule Synthesis

The choice of alginate and dextran concentrations is based on data from the literature [23,24,25,26].

**Figure 2:**
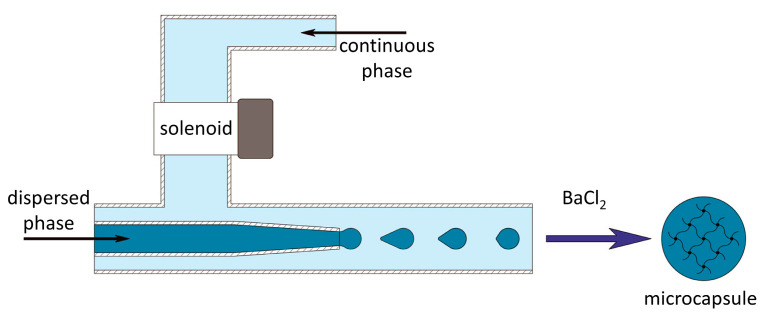
Schematic image of the microfluidic device.

To create and test the microcapsules, a microfluidic setup was developed. It operates based on well-known principles of microfluidics [27]. To create the alginate microcapsules, the external influence of the dispersed phase on the flow of the continuous phase is used [28] (Figure 2). The operating conditions of the microfluidic setup were chosen so that microcapsules with an average size of 496 ± 46 µm were formed.

**Figure 3:**
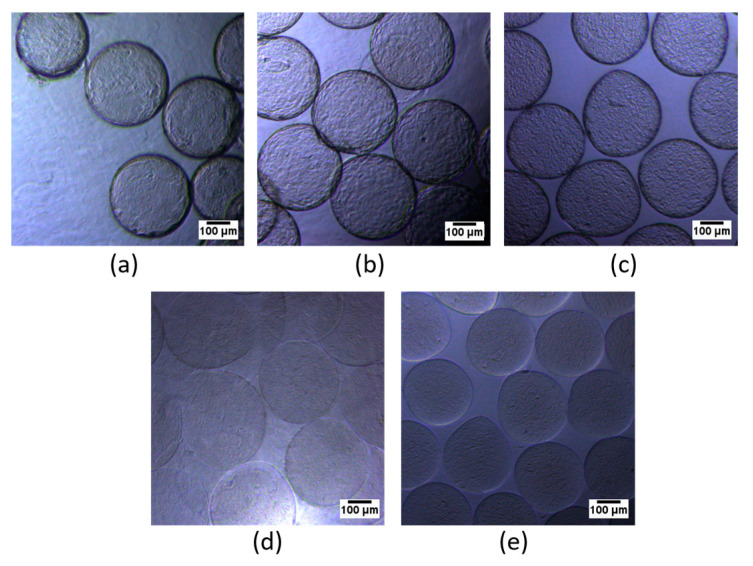
Bright-field images of alginate microcapsules incubated in various cationic PMETAC solutions: (**а**) 1%, (**b**) 0.4%, (**c**) 0.1%, (**d**) 0.01%, (**e**) 0% (Scale bar 100 μm).

To increase the stability of the alginate microcapsules, they were coated with the cationic polymer PMETAC using solutions of the following concentrations: 0%; 0.01%; 0.1%; 0.5%; 0.4%; and 1%. Visually, an increase in PMETAC concentration correlated with increased thickness and induration of the microcapsule shell (Figure 3). Subsequently, the microcapsules were covered with an additional layer of alginate to increase their biocompatibility.

### 3.3. Permeability Measurements

The permeability of such microcapsules is one of the most important parameters for the immunoisolation of the encapsulated cells. The permeability of these membranes depends upon the porosity and the ability of the membrane to retain molecules of a particular size and is expressed as the molecular weight cut-off (MWCO) [29]. Conceptually, immunoisolation microcapsules require pore sizes that allow the exchange of glucose (180 Da; 0.4 nm) and insulin (5.8 kDa; 2.05 nm), while blocking inflammatory cells (~10 μm), immunoglobulins (150–910 kDa; 5.3–12.6 nm), and the complement components C3 and C5 (~190 kDa; 5.3 nm) [30].

The permeability of alginate microcapsules coated with PMETAC to 36 and 120 kDa FITC-lectins was tested in vitro. The number of fluorescent lectins that penetrated into the alginate beads were counted in the brightest part of the image just inside the boundaries of the microcapsules and reported for an area of 10,000 pixels^2^. Upon examination after incubation with FITC-lectins, unencapsulated naked islets contained 39 ± 14 fluorescent lectins/10 000 p^2^, while empty microcapsules contained 2.5 ± 2 fluorescent lectins/10,000 p^2^, which indicated that the presence of islet tissue was necessary for fluorescence activity to occur within the microcapsules. The microcapsule was considered permeable to lectins if the median fluorescence exceeded 5 lectins/10,000 p^2^, since this value was the maximum found in empty microcapsules.

**Figure 4:**
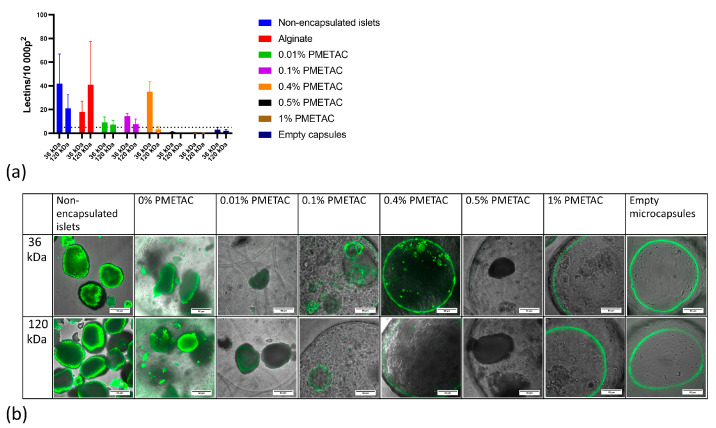
Comparison of the permeability of microcapsules coated using different concentrations of PMETAC. (**a**) Graph, (**b**) confocal fluorescence image (Scale bar 50 μm).

We have determined that the concentration of the alginate polymer coating affects the permeability of the microcapsule (Figure 4). With increasing polymer concentration, the permeability of the microcapsules decreases. Alginate microcapsules coated with 1% and 0.5% PMETAC are impermeable to low-molecular weight lectins, so the diffusion of nutrients can be difficult, making such microcapsules unsuitable for islet encapsulation. By contrast, alginate microcapsules that are not coated or coated with 0.01% or 0.1% PMETAC are permeable to 120 kDa lectins, so such microcapsules will not isolate the islet from the immune system and are therefore not suitable for transplantation. Alginate microcapsules coated with 0.4% PMETAC are permeable to low-molecular weight lectins and impermeable to high-molecular weight lectins. Thus, in our tests, we have demonstrated that microcapsules with 0.4% PMETAC present optimum results in terms of permeability—they are selectively permeable and suitable for islet encapsulation for the purpose of immunoisolation.

### 3.4. Microcapsule Stability Study

To determine the mechanical and thermal stability of the microcapsules, possible variations in microcapsule diameters and in the number of broken structures were determined under different stress conditions.

To study the stability of PMETAC-coated alginate microcapsules, we tested several variants of microcapsule variants. Alginate microcapsules were used as controls, and corresponding microcapsules coated with 1%, 0.1%, or 0.01% PMETAC allowed us to study changes in their stability with changes in polymer concentration, while alginate microcapsules coated with 0.4% PMETAC provided the means to investigate their stability, since these microcapsules are appropriately selectively permeable.

#### 3.4.1. Evaluation of Osmotic Stability

**Figure 5:**
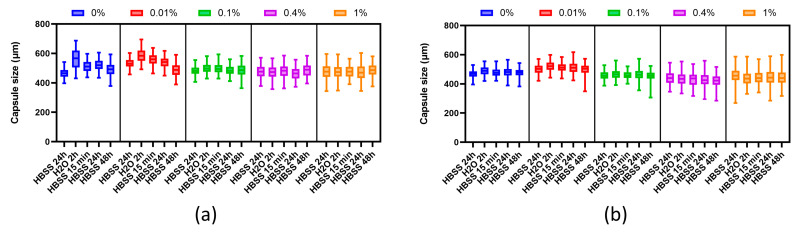
Graph of changes in the size of microcapsules coated with different concentrations of PMETAC in the test for osmotic stability carried out at (**a**) 2 days and (**b**) 7 days.

We performed the analyses 2 days after microcapsule fabrication because that is the approximate time-point when they would typically be implanted when used in clinical settings (Figure 5a). Tests were also performed at 7 days post-fabrication to ensure there was no loss of microcapsule mechanical integrity (Figure 5b).

**Figure 6:**
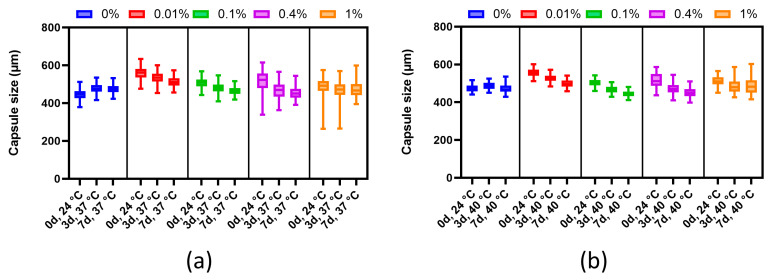
Graph of сhanges in the size of microcapsules coated with different concentrations of PMETAC in the test for thermal stability: (**a**) 37 °C and (**b**) 40 °C.

We first assessed the extent of microcapsule swelling in response to an osmotic pressure test. Initially, the microcapsules were held for 24 h in Hank’s solution (HBSS), which has a physiological osmotic pressure (270–305 mOsm). The size at the end of this stage is represented by HBSS 24 h in Figure 6. Placing the microcapsules in a hypotonic environment (water, 0 mOsm) for 2 h resulted in the entry of water, driving an increase in their volume (HBSS 2 h). In the second stage, the return of the microcapsules to an isotonic environment led to either short- or long-term restoration of the microcapsule diameter after the removal of this osmotic stress. The different rates of recovery of the original microcapsule size can be interpreted using Burger’s model. Such a test probes bulk microcapsule resistance to deformation and allows for distinction between elastic recovery and plastic deformation. Volumetric microcapsule stability is highly relevant to the success of encapsulated islet grafts because uncontrolled swelling and irreversible deformation lead to a loss of islet immunoprotection.

All microcapsules used passed the osmotic stress test. During the test on the second day, the microcapsules resisted the osmotic forces but swelled by up to 30%. Nevertheless, reinforcement of the alginate microcapsules with PMETAC resulted in higher stability. When placing samples in an isotonic medium, the size change of the alginate microcapsules coated with 0.01% PMETAC, 0.1% PMETAC, 0.4% PMETAC, and 1% PMETAC was 10.6%, 3.5%, 1.0%, and 0.6%, respectively.

After the continuous stress phase, the osmotic pressure was removed. Measurement of the microcapsule diameter at 15 min (HBSS 15 min) post-stress allowed us to identify the immediate elastic contribution to microcapsule recovery. The 1-day (HBSS 24 h) uncoated alginates and 0.01% PMETAC-coated microcapsules only recovered by 50% strain after stress removal, indicating a significant viscous retardation of sample recovery. At greater PMETAC thicknesses, the contribution of the elastic properties was increased. Alginate microcapsules coated with PMETAC at a concentration of more than 0.1% fully recovered within the first 15 min, illustrating their elastic properties.

We continued monitoring microcapsule recovery over time to describe the complete viscoelastic response. The alginate-only microcapsules continued to recover slowly to 5% strain, demonstrating a viscously damped elastic response compared to the “instantaneous” elastic response of the PMETAC-coated alginate microcapsules. Two days (HBSS 48 h) after the removal of osmotic stress, the alginate microcapsules coated with 0.01% PMETAC show a reduction in size, which was associated with the destruction and rupture of the microcapsule shell.

Interestingly, all of the microcapsules displayed improved mechanical properties when tested after 7 days. This increased elasticity has been reported before and may be due to spontaneous, thermodynamically favored alginate chain rearrangements or the sequestration of additional calcium [31].

#### 3.4.2. Evaluation of Thermal Stability

The thermal stability analysis was performed in accordance with the methodology of [31] with minor modifications. A quantitative assessment of the change in the diameter of the microcapsule during long-term (3 and 7 days) incubation at physiological and elevated temperatures in osmotically balanced solutions showed that the alginate-only microcapsules tended to retain their diameter (average ratio of the final diameter to the initial one: 0%—37 °C, 1.06; 0%—40 °C, 0.99). Modified alginate microcapsules coated with 0.01%, 0.1%, 0.4%, or 1% PMETAC showed the dependence of shrinkage on the mass fraction of the polymer, and the absence of any pronounced effect of temperature conditions (Figure 6). At 37 °C and 40 °C, a slight shrinkage compared to alginate-only microcapsules was demonstrated by microcapsules coated with 1% polymer (average ratio of final diameter to initial: 37 °C, 0.97, *p* = 0.0000, 40 °С, 0.95, *p* = 0.0000). The 0.1% and 0.01% polymer-coated microcapsules showed similar changes in diameter (average ratio of final diameter to initial: 37 °C, 0.91, *p* = 0.0000, 40 °С, 0.89, *p* = 0.0000). We observed that all PMETAC-coated microcapsules were larger than the control alginate-only microcapsules. As it is known that the viscosity of alginate decreases with increasing temperature [32], it is probable that the application of only a thin coating of PMETAC allowed the alginate to swell, but as there was a gradual exit of some of it through the pores, this led to shrinkage of the microcapsules. The dense coating of 1% PMETAC most likely blocked the free exit of the less viscous alginate from the microcapsule, leading to their expansion. Nevertheless, the decrease in the size of the microcapsules did not significantly impact the pore size. Mathematical analysis revealed (data not provided) that despite the potential change in permeability from 35 kDa to 30–32 kDa, nutrients and insulin could still permeate through the microcapsules.

#### 3.4.3. Stability of Microcapsules under Culture Conditions

**Figure 7:**
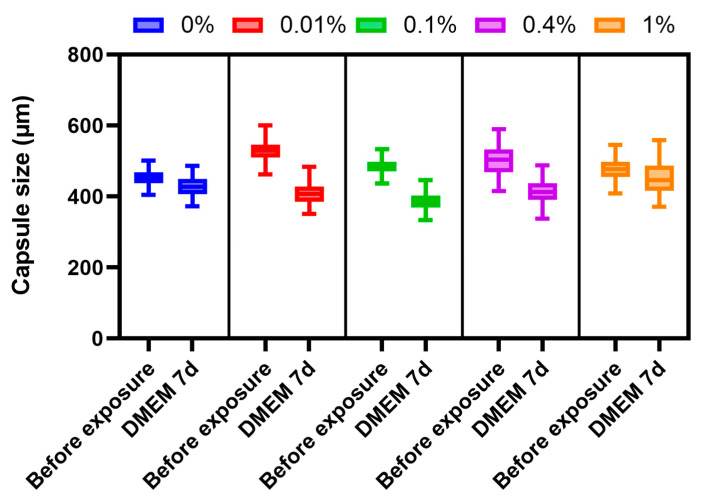
Graph of сhanges in the size of microcapsules coated using different PMETAC concentrations when tested for stability of microcapsules under culture conditions.

For studying changes in the stability of microcapsules under cell culture conditions in DMEM, we relied on the study by [33], but changed the duration of the experiment. A quantitative assessment of changes in the diameter of the microcapsules showed that alginate microcapsules with different PMETAC coatings tended to decrease in diameter over a period of 7 days under culture conditions, with a dependence of the shrinkage on the mass fraction of the polymer (Figure 7). The data obtained may be related to the characteristics of alginate microcapsules [31]. The smallest shrinkage was observed in microcapsules coated with 1% polymer (average ratio of the final diameter to the initial one: 0.937, *p* ≤ 0.001) and microcapsules coated with 0.4% polymer (average ratio of the final diameter to the initial one: 0.821, *p* ≤ 0.001). Such a change in the diameter range demonstrates the inverse relationship between the elasticity of the microcapsules and the amount of PMETAC. However, these small changes in capsule sizes do not make significant alterations to the permeability of the capsules. Thus, all capsules are stable under culture conditions.

#### 3.4.4. Multi-Wash Microcapsule Stability Test

**Figure 8:**
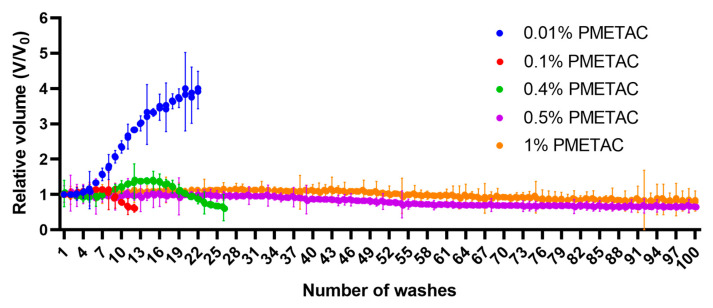
Graph of сhanges in the volume of microcapsules, coated using different concentrations of PMETAC, during the multi-wash microcapsule stability test.

In this test, the stability of the alginate microcapsules was assessed by repeatedly washing them in isotonic saline solution, with a determination of the volume occupied by the microcapsules after each wash. The number of washes of the microcapsules before their dissolution or destruction was taken as the indicator of their stability. The results of these stability studies are shown in Figure 8.

**Figure 9:**
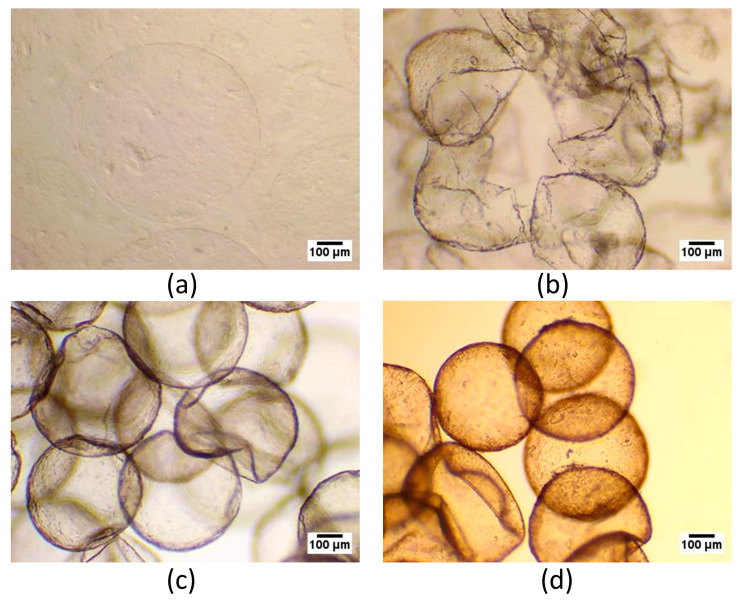
Bright-field images of alginate microcapsules, previously incubated in polymer (PMETAC) solutions of different concentrations, (**а**) 0.01%, (**b**) 0.1%, (**c**) 0.4%, (**d**) 1%, after repeated washing in saline (Scale bar 100 μm).

It was found that microcapsules without any additional polymer coating began to swell after the fifth wash. During washing, the volume (V) occupied by the microcapsules increased to almost four times the original volume (V_0_) (Figure 9). In this case, the microcapsules became visually poorly distinguishable. During the washing of hydrogel microcapsules in physiological saline, barium ions are reversibly replaced by sodium ions, thereby reducing the degree of microcapsule cross-linking. It was established that microcapsules without a polymer membrane completely dissolved after the twentieth wash.

When washed in saline, microcapsules incubated in a solution with 0.01 wt.% PMETAC increased in volume by no more than 15%. After the sixth wash, the microcapsules became poorly distinguishable visually, and with further washing in physiological saline, their volume decreased sharply. Probably, by this time, the barium alginate had dissolved so much that the thin polymer shell became torn, allowing the microcapsules to disintegrate.

If the concentration of PMETAC was increased, a denser shell was formed. When using a 0.1 wt.% polymer solution, the volume occupied by the microcapsules first increased, which can be explained by the swelling of the microcapsules, and then decreased after the fifteenth washing, which is associated with the destruction of the microcapsules. Only the remains of torn, crumpled polymer shells, as well as deformed and torn microcapsules, could be observed under a microscope. At a concentration of PMETAC of 0.4%, the volume occupied by the microcapsules steadily decreased, and after one hundred washes, 70% of the microcapsules still remained. When washed in physiological saline, microcapsules treated in 1% polymer solution did not change visually. Initially, the volume occupied by the microcapsules increased by 10%, and then decreased after the fortieth wash. After one hundred washes, the microcapsules occupied about 80% of their original volume.

Thus, incubation of microcapsules in PMETAC can significantly increase their resistance to washing in saline. In this case, the stability of the microcapsules increased with increased concentrations of the cationic polymer.

Based on the results of all the permeability and stability tests, alginate microcapsules coated with 0.4% PMETAC were selected for further work (Table 1). They had demonstrated the necessary properties, including both permeability and stability, by passing most tests. The increase in stability of such microcapsules, resulting from increasing the concentration of PMETAC, is inversely proportional to its effect on permeability. Thus, alginate microcapsules coated with PMETAC at a concentration of 0.4% are optimally stable, provided that the permeability to low-molecular weight compounds is maintained.

#### 3.4.5. Viability and Functional Activity of Encapsulated Islets

**Figure 10:**
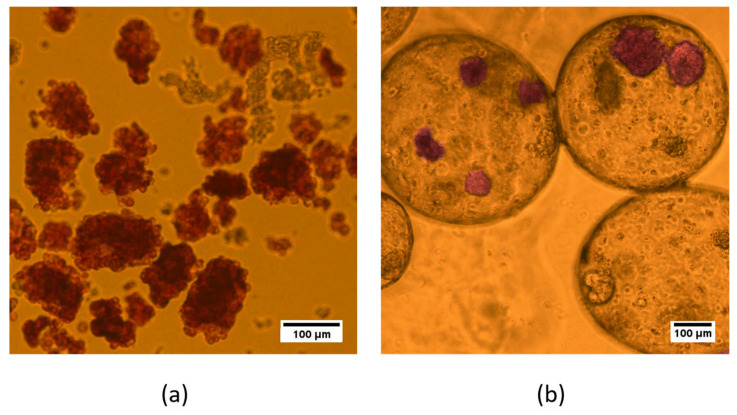
Bright-field images of islets of Langerhans stained with dithizone. (**a**) Unencapsulated, (**b**) encapsulated (Scale bar 100 μm).

Islets were isolated from Wistar rats weighing 300 ± 50 g using the standard protocol with our modifications. The average yield was 400 ± 100 islets from each pancreas. Figure 10 shows freshly isolated rodent islets after purification. Purity in excess of 80% is routinely attained (Figure 10). The nature of the isolated cells was confirmed by specific staining with dithizone. A total of about 30,000 islets of Langerhans were obtained for use in our experiments.

**Figure 11:**
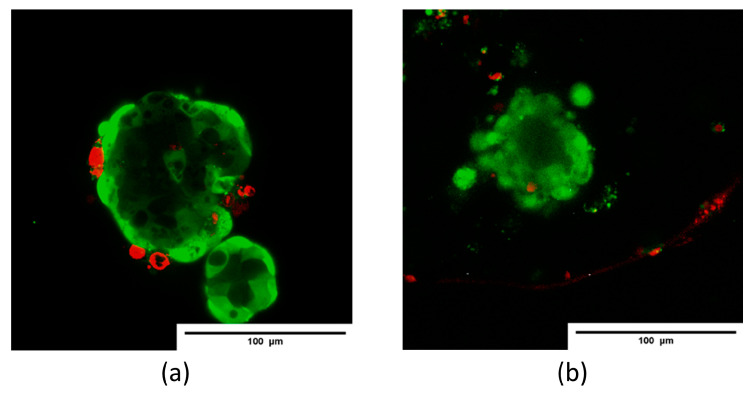
Confocal fluorescence images showing islets (**a**) without and (**b**) with encapsulation, labeled with viability stains Calcein AM (green) and propidium iodide (red). (Scale bar 100 μm).

Dithizone staining was used to identify the quality of islets after encapsulation. DTZ readily complexes with transition metals, including zinc, and can identify zinc-replete cells such as islets. Zinc is an important element present within the insulin granules of the β-cells. Zinc ions are essential in maintaining the structure and integrity of insulin molecules within β-cells and are secreted with insulin [34,35]. Thus, after encapsulation, the islets maintained their morphology and the ability to synthesize insulin (Figure 11).

**Figure 12:**
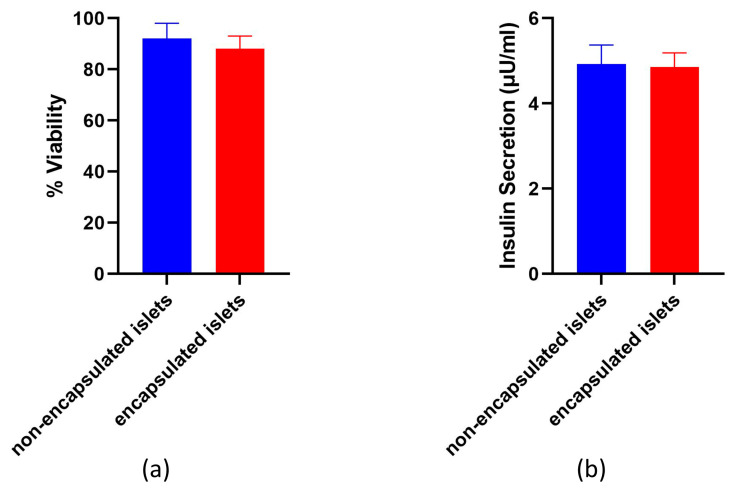
Graph of changes in the (**a**) viability and (**b**) functional activity of pancreatic islets before and after encapsulation.

After 24 h of culture, the percentage of viable cells in each non-encapsulated and encapsulated islet was analyzed to determine the effect of encapsulation on the viability of the islets of Langerhans. As seen in the representative live/dead images (Figure 12), the encapsulated islets did not show a significant reduction in the number of viable cells compared to islets cultured under standard culture conditions. Quantitative image analysis of the live/dead results did not show (Figure 12a) a statistically significant difference in the percentage of viable cells after encapsulation (85.66 ± 1.74%, *p* = 0.0828) compared to non-encapsulated islets (88.64 ± 1.56%).

An enzyme-linked immunosorbent assay (ELISA) was conducted after a 24 h period of incubation and showed that the islets secrete insulin. In one day of incubation in the culture medium, 350 islets produced 4.83 ± 0.26 μU/mL of insulin. After encapsulation, insulin synthesis by the islets remained quite high at 4.64 ± 0.29 μU/mL compared to non-encapsulated islets (Figure 12b). There was no statistically significant difference in the level of insulin synthesis in OL before and after encapsulation (*p* = 0.08), indicating the preservation of natural insulin secretion.

## 4. Discussion

In this study, we developed and tested a new microcapsule for islet encapsulation to address insulin deficiency. Our approach is innovative in two key ways. First, we applied the polymer PMETAC, which has not been previously used specifically for encapsulating islets of Langerhans. Second, unlike previous methods where PMETAC was combined with copolymers, we utilized PMETAC independently, creating a simpler and more efficient microcapsule.

By combining alginate with PMETAC, we optimized the capsule’s composition and preparation process. PMETAC’s non-cytotoxicity and excellent biocompatibility make it an ideal alternative to traditional anionic coatings. This simplified approach not only reduces production costs but also enhances the microcapsules’ potential for broader clinical application.

Alginate microcapsules coated using various concentrations of PMETAC have been tested for their permeability, stability, and cytotoxicity. The best result was shown by alginate microcapsules coated with 0.4% PMETAC. The islets remained viable and functionally active. Microcapsules coated using 0.4% polymer have the necessary permeability, are resistant to deformation by osmotic pressure, are thermally stable, change in volume by less than 15% when incubated in culture medium, and do not collapse when washed with physiological media.

The core of the microcapsule we worked with was alginate. Alginate is one of the well-studied natural polysaccharides that are most commonly used for the encapsulation of biological objects. Alginate hydrogels exhibit good biocompatibility and are often used to create shells around pancreatic islets of Langerhans [32]. However, in other studies, alginate microcapsules have shown only limited success where their long-term survival is needed. Alginate microcapsules are unstable and rapidly biodegrade [36] and are also sensitive to chelating agents, such as phosphate and citrate, and gel-degrading agents such as sodium and magnesium ions. In physiological solution, such replacement of ions results in osmotic swelling of the microcapsules, inevitably leading to increased pore size, destabilization, and rupture of the gel. Thus, the high porosity of the alginate network has promoted the development of different coating techniques [37]. In our studies, we found that uncoated alginate microcapsules were also unstable and permeable to high-molecular weight compounds, which limited their effectiveness.

Traditionally, poly-L-lysine (PLL) and poly-L-ornithine (PLO) have been used to strengthen the surfaces of alginate microcapsules. These cationic polymers can form a shell around such microcapsules due to ionic bonding between unbound calcium/barium and the carboxylate alginate anions. Although both capsule types demonstrated the required permeability, it was found that PLO capsules were much more stable and swelled less in saline than PLL capsules [7]. Our PMETAC-coated capsules demonstrated a similar improved stability compared to PLL capsules, similar to the stability observed with PLO capsules. In the next step, a layer of alginate is then deposited on the PLL or PLO layer. The outer alginate shell seals the cationic PLL from the tissues of the microcapsule recipient and thus makes the microcapsules biocompatible. Although microcapsules with this type of structure meet many of the requirements for cell immunoisolation, they have insufficient strength when implanted in large animals [38].

Recent work has demonstrated the value of PMETAC for biocompatible implants. The application of PMETAC forms coatings that can be used successfully with biomaterials [39]. Initially, PMETAC was used as a copolymer in the creation of alginate microcapsules for cell culture encapsulation [18,40]. The resulting microcapsules provided sufficient permeability and indicated that this polymer may be suitable for encapsulating the islet apparatus.

One of the most important parameters of microcapsules used for cell immunoisolation is their selective permeability. While the microcapsule must protect cells from destruction by the immune system, it must simultaneously permit the permeation of oxygen and biological compounds required for the viability and normal functioning of the cells inside. The final permeability of the microcapsules can depend on the conditions for their synthesis, the molecular weight of the components of the microcapsules, the duration of coating of the alginate with PMETAC, and its concentration in the coating solution [41]. For polymers such as poly-L-lysine and poly-L-ornithine, a concentration of 0.05% is considered sufficient to create immunoisolation of the content of the microcapsules [42]. In this work, we have shown the dependence of coating formation on PMETAC concentration and the molecular weight permeability cut-off of PMETAC-coated alginate microcapsules. We have created microcapsules that ensure the efficient diffusion of insulin molecules and prevent the diffusion of larger molecules. After being exposed to PMETAC polymers at a concentration of 0.4% for 10 min, alginate microcapsules develop the required permeability. We have proven that our microcapsules do not allow molecules larger than 120 kDa to pass, which is in line with other studies [11].

The successful application of such microcapsules is dependent on their durability and stability. The microcapsules should be mechanically able to withstand internal sheering forces without compromising their permselectivity or biocompatibility. To address this key requirement, the microcapsules must withstand the internal sheer stresses and osmotic pressures generated both during microcapsule formation and implantation [43]. Under the influence of low osmotic pressure conditions in the medium on the day after synthesis, the microcapsules we obtained increased by no more than 20–30% in volume. In other studies, researchers have also demonstrated the stability of their microcapsules, there being no more than a 30% dimensional change after exposing the microcapsules to water [11]. With increased PMETAC concentration, the stability of the microcapsules also increases. Our results indicated that microcapsules using 0.4% PMETAC were both resistant to deformation by changing osmotic pressure and showed elastic properties. We followed the protocol for testing the osmotic stability of alginate microcapsules, but the difference was that the capsules in this study were reinforced with polyethylene glycol (MicroMix) [31]. This study showed that MicroMix capsules demonstrated improved resistance to bulk osmotic stress compared to alginate-only capsules (ALG-only). Similarly, our PMETAC-coated capsules demonstrated osmotic stability comparable to MicroMix capsules. Moreover, we observed that higher concentrations of PMETAC further enhanced the stability of the capsules. Thus, we found that the osmotic stability of PMETAC-coated capsules is similar to that of MicroMix capsules.

Microcapsules must be able to withstand both the temperature range within the recipient’s body and the conditions used for cell incubation before transplantation. To analyze such thermal stability, we increased the microcapsule incubation time compared to the works of other authors and found that there was a decrease in microcapsule diameter when using PMETAC at a concentration below 1%. However, this reduction in microcapsule size did not critically affect the pore size. Mathematical analysis showed (data not included) that although the permeability of the microcapsules might change from 35 kDa to 30–32 kDa, nutrients and insulin could still continue to diffuse through the microcapsule. Analysis of the stability of microcapsules under long-term culture conditions showed a decrease in the sizes of alginate microcapsules coated with PMETAC, with a dependence of the shrinkage on the mass fraction of the polymer. Our findings indicate that PMETAC-coated capsules have mechanical properties comparable to PolyLN-Biodritin and LN-Biodritin capsules in similar tests [33]. Thus, microcapsules coated using 0.4% PMETAC exhibit sufficient long-term stability when compared to other polymers.

A test model using multiple rinsings with saline indicated the resistance of alginate microcapsules coated with 0.4% PMETAC to such an impact. This test showed that the change in the size of the microcapsules is limited. Thus, we obtained microcapsules by using 0.4% PMETAC, which, despite a 5% reduction in their size, maintained their shape. This test suggested that these microcapsules should show long-term stability in vivo. Together, these tests demonstrated that the initial gel strength is largely due to the alginate gel cores, while the long-term strength is solely due to the polymer shells [44]. Increasing the concentration of PMETAC increased the stability of the microcapsules but decreased their permeability. These data correlate with the results of alginate microcapsules coated with poly-L-lysine. An increase in the thickness of the poly-L-lysine polymer coating from 35 µm to 120 µm has been found to cause changes in their resistance to loads from 0.1 ± 0.01 g/microcapsule to 2 ± 0.2 g/microcapsule and a change in permeability from 150 kDa to 40 kDa [45]. The use of PMETAC at a concentration of 0.4% leads to the formation of microcapsules that are optimal in terms of stability and permeability.

The viability and functional activity of encapsulated islets are key to successful transplantation. The encapsulation process can potentially stress insulin-producing cells, causing them to lose functionality and their properties. The cells may be affected by the mechanical effects of encapsulation, the temporary presence of a viscous alginate around them, or the cytotoxic properties of the encapsulation solutions. Therefore, it is essential to evaluate the impact of encapsulation systems on the islets of Langerhans. This is supported by the results of a study that demonstrated that encapsulation in Alginate-Ca^2+^/Ba^2+^ microbeads did not compromise the viability or insulin secretion of human islets during glucose stimulation [20]. Moreover, certain microcapsules can enhance the viability and functional activity of islets by incorporating additional cells or molecules. When mesenchymal stem cells and arg-gly-asp tripeptides were added to microcapsules, this resulted in a 29.9% ± 5.7% increase in islet viability (*p* = 0.02) and a two-fold increase in functional activity compared to unencapsulated islets (*p* = 0.017) [46]. Cells that were encapsulated in an alginate microcapsule coated using 0.4% PMETAC remained viable and functionally active, thus indicating that such a microcapsule has a minimal effect on the cells.

Therefore, microcapsules coated using 0.4% PMETAC have the necessary permeability, are resistant to osmotic deformation, are thermally stable, and change volume by less than 15% when incubated in culture medium. Furthermore, the repeated washing of such microcapsules does not destroy them, and the microcapsules, themselves, do not have a cytotoxic effect on the islet cells. Changes in microcapsule size during long-term storage can be avoided by using PMETAC at higher concentrations. However, an increase in the mass fraction of PMETAC leads to a sharp decrease in permeability. Another option for solving the problem of microcapsule shrinkage is the use of an additional layer to create a strong bond between the PMETAC and another polymer, for example, an anionic polymer. In this way, the stiffness of the microcapsule membrane could be increased, although the extent of selective permeability would need to be reassessed. Our unprecedented study of microcapsules based on alginate, coated only with PMETAC, has indicated that the use of a 0.4% coating does exhibit appropriate long-term stability.

Alginate microcapsules coated using 0.4% PMETAC are therefore promising for the development of an encapsulating system for the immunoisolation of pancreatic islets. For the further use of such microcapsules, additional studies are needed to investigate their properties, including their biocompatibility.

## 5. Conclusions

Our study was the first to analyze alginate microcapsules coated with PMETAC. For this, a special microfluidic setup was created. The use of PMETAC can improve the biocompatibility and durability of the capsules, marking a significant advancement in islet encapsulation techniques. Moreover, our innovative approach combines alginate with PMETAC to create microcapsules containing islets of Langerhans. This development not only simplifies the composition of starting materials but also optimizes the preparation process, reduces costs, and enhances the potential for widespread clinical use. Polymer-coated microcapsules with 0.01–1% PMETAC were tested. It was shown that with an increase in the concentration of the polymer, the stability of the microcapsules increases in tests for osmotic and thermal stability and under cell culture conditions, as well as with repeated washing with a physiological medium. The microcapsule coatings made using 0.4% PMETAC showed the most promising results as they were stable in the above tests while having the required permeability. Namely, they are permeable to low-molecular weight compounds (up to 35 kDa) and impermeable to high-molecular weight compounds (120 kDa and above). Such permeability is necessary for immunoisolation. When encapsulated, the pancreatic islets remain viable and functionally active. Thus, alginate microcapsules coated using 0.4% PMETAC are suitable for further animal testing. It should also be possible to refine their properties by adding further microcapsule layers.

## Figures and Tables

**Table 1:** Evaluation of the success, during testing, of alginate microcapsules coated by exposure to different concentrations of PMETAC. Plus—test passed, Minus—test failed.

	0% PMETAC	0.01% PMETAC	0.1% PMETAC	0.4% PMETAC	1% PMETAC
*Permeability measurements*	−	−	−	+	−
*Osmotic stability*	−	−	+	+	+
*Thermal stability*	+	+	+	+	+
*Stability under culture conditions*	+	+	+	+	+
*Multi-wash microcapsule stability*	−	−	−	+	+

## Data Availability

Data available on request from the corresponding author.
